# Navigating the ICI Combination Treatment Journey: Patterns of Response and Progression to First-Line ICI-Based Combination Treatment in Metastatic Renal Cell Carcinoma

**DOI:** 10.3390/jcm13020307

**Published:** 2024-01-05

**Authors:** Alessandro Samuelly, Rosario Francesco Di Stefano, Fabio Turco, Marco Donatello Delcuratolo, Chiara Pisano, Isabella Saporita, Mariangela Calabrese, Federica Maria Carfì, Marcello Tucci, Consuelo Buttigliero

**Affiliations:** 1 Department of Medical Oncology, University of Turin, San Luigi Gonzaga Hospital, 10043 Orbassano, Italy; alessandro.samuelly@unito.it (A.S.); fabio.turco523@edu.unito.it (F.T.); isabella.saporita@unito.it (I.S.); mariangela.calabrese@unito.it (M.C.);; 2 Department of Medical Oncology, S. Croce e Carle Hospital, 12100 Cuneo, Italy; pisano.c@ospedale.cuneo.it; 3 Department of Medical Oncology, Cardinal Massaia Hospital, 14100 Asti, Italy

**Keywords:** kidney cancer, metastatic clear cell renal cell carcinoma, immuno-oncology, treatment, immune checkpoint inhibitors, tyrosine kinase inhibitors

## Abstract

The use of immune checkpoint inhibitors (ICIs) in combination with tyrosine kinase inhibitors or other ICIs has significantly improved the prognosis for patients with mccRCC. This marks a major milestone in the treatment of mccRCC. Nonetheless, most patients will discontinue first-line therapy. In this narrative review, we analyze the different patterns of treatment discontinuation in the four pivotal phase III trials that have shown an improvement in overall survival in mccRCC first-line therapy, starting from 1 January 2017 to 1 June 2023. We highlight the different discontinuation scenarios and their influences on subsequent treatment options, aiming to provide more data to clinicians to navigate a complex decision-making process through a narrative review approach. We have identified several causes for discontinuations for patients treated with ICI-based combinations, such as interruption for drug-related adverse events, ICI treatment completion, treatment discontinuation due to complete response or maximum clinical benefit, or due to progression (pseudoprogression, systemic progression, and oligoprogression); for each case, an extensive analysis of the trials and current medical review has been conducted.

## 1. Introduction

Kidney cancer is one of the most common urological cancers, accounting for 2.2% of new cancer diagnoses and 1.8% of cancer deaths worldwide [1], with a higher burden falling on North American and Western European populations [2]. In total, 1 in 46 citizens of the United States will develop an invasive renal or pelvis cancer during his/her lifetime, a burden that is slowly increasing from 1 in 49 in 2014 [3,4]. Renal cell carcinoma (RCC) is the most common type of kidney cancer, with the clear cell subtype (ccRCC) accounting for about 70% of all RCC cases [5]. The 5-year relative survival rate across all risk groups for metastatic RCC in the SEER database from 2011 to 2017 was approximately 14% [3]. With the approval and the widespread adoption of combination treatments (immuno oncology (IO) drugs in combination with other IO or with tyrosine kinase inhibitors) in the first-line setting, the outcomes are improving. Survival analyses of a global cohort of patients stratified according to the International Metastatic RCC Database Consortium (IMDC) risk criteria, receiving first-line immuno-combination treatments, presented a more recent and optimistic overview, as IMDC good-risk patients exhibited a median overall survival that was not reached (NR, 95% CI NR–NR), while those in the intermediate-risk group showed a median overall survival of 55.7 months (95% CI 28.4–60.8), and individuals classified as poor risk had a median overall survival of 19.2 months (95% CI 12.5–32.7), dramatically improving the previous outcomes [6].

With the introduction of ICI-based combinations in the first-line setting, clinicians face new challenges regarding the choice of treatment sequencing, as there are a variety of potential outcomes and different scenarios that may necessitate distinct management strategies, such as continuing with ICI monotherapy, continuing the same therapy, using local therapy, or switching to alternative treatments. This review aims to explore the various outcomes following first-line systemic therapy for mccRCC and their best management.

## 2. First-Line Therapy for Clear Cell Histology

**Figure 1:**
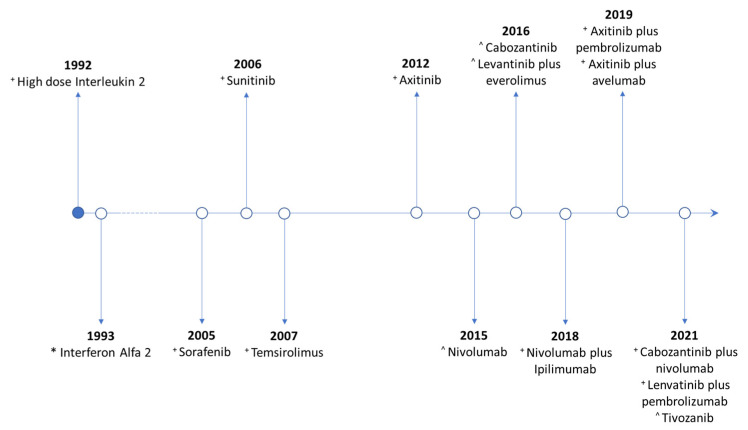
Timeline for mRCC treatment based on FDA approvals. * Interferon alfa-2a never received approval from the FDA as a monotherapy; in 1993, the results of the first trial were published. It has been inserted in the timeline as it was used as the comparison group for sunitinib and temsirolimus in the respective registration trials. + approved for the frontline treatment. ^ approved for subsequent lines of therapy.

There have been considerable improvements in the overall survival (OS) of metastatic renal cell carcinoma (mRCC) in recent decades; in Figure 1 we summarised the main FDA drug approvals for mRCC treatment. In the 1990s, the use of high-dose interleukin-2 (IL-2) led to up to 8% of patients achieving complete responses, most of whom would have a sustained response for years after discontinuation but with a low objective response rate (ORR), at around 20%, and the development of frequent and often severe toxicities. Moreover, patients often did not respond to this treatment and progressed quickly [7]. The toxicity profile and the inability to identify patients who could benefit from therapy hampered wide HD-IL2 adoption [8].

Interferon alfa-2a (IFN-a) had a better toxicity profile than the first cytokine but was less effective in treating mRCC with lower response rates and shorter duration of response [9]. IFN-a was only approved by the FDA in 2009 for mRCC, in combination with bevacizumab, but never as monotherapy. However, it was used as a comparison in the pivotal sunitinib trial based on evidence of its efficacy in previous trials and widespread adoption [10]. 

It was more than a decade after IL-2 before the new class of protein kinase inhibitors entered clinical practice. Targeted therapies for mRCC include mTOR inhibitors (temsirolimus, everolimus) and Multitargeted Tyrosine Kinase Inhibitors (TKIs), most of which are mainly VEGFR inhibitors (sorafenib, sunitinib, pazopanib, axitinib, lenvatinib, and cabozantinib). In the early 2000s, the arrival of sunitinib, the first tyrosine kinase inhibitor, improved objective response rates (ORR) to around 30% and had a better toxicity profile in comparison with cytokines [10,11]. Yet, complete responses were less frequent, at around 1% [12], most of whom would relapse after treatment discontinuation [13].

For over a decade, sunitinib or pazopanib, and to a lesser extent bevacizumab plus INF-alpha, were considered the standard of care as first-line treatment for patients with mRCC.

In 2016, cabozantinib was approved by the European Medicines Agency (EMA) as a first-line treatment for mRCC patients with intermediate or poor IIMDC risk based on the results of the phase II CABOSUN trial [14].

In 2015, nivolumab was the first therapy belonging to the class of ICIs to demonstrate efficacy in the treatment of mRCC. In the phase III CheckMate 025 trial, nivolumab improved OS compared with everolimus in 821 pre-treated patients (median OS: 25.8 versus 19.7, HR 0.73, 95% confidence interval (CI), 0.62–0.85; *p* < 0.0001) [15,16]. ICIs had considerable benefits compared to the previous generation of immunotherapies, with less toxicity, higher and more durable responses, and, as it would be later discovered, the possibility of combining them with kinase inhibitors without a substantial increase in toxicity.

In recent years, four phase 3 trials in mRCC in the first-line setting demonstrated an improved OS with ICI-based combination (ICI-ICI and ICI-TKI) compared to TKI monotherapy, initiating what can be called the “golden age” of kidney cancer.

In 2018, the combination of nivolumab and ipilimumab, a monoclonal antibody inhibiting the CTLA-4 receptor in T cells, was approved for the frontline setting following the CheckMate 214 [17], demonstrating a benefit over sunitinib in OS for IMDC criteria intermediate-/poor-risk patients (median OS: 48.1 versus 26.6 months; HR 0.65, 95% CI 0.54–0.78), a complete response rate previously unseen of 9%, and an ORR of 41.9%, weighted against a moderate increase in toxicity, even if treatment-related adverse events led to the discontinuation in 22% of patients (against 12% for sunitinib). No differences in OS were observed in the favorable-risk patients (HR 0.93, 95% CI 0.62–1.40). A recent update with five-year follow-up shows ongoing benefits for the immune combination (median OS not reached in IMDC intermediate-/poor-risk patients) and a further small increase in complete responses, reaching 12% in the ITT analysis across all risk groups [18].

ICI-ICI and ICI-TKI combinations have become the standard of care in the frontline setting, especially for IMDC intermediate and poor-risk patients [19], whereas for IMDC favorable-risk disease, there has yet to be an improvement in OS over TKI monotherapy [20]. The addition of PD-(L)1 inhibitors to target therapies provides a higher objective response rate (ORR) and durable responses, with complete response rates of up to 8–16% in pivotal trials (ref. Table 1). Even so, most patients relapse.

The **Checkmate 214** was the first phase III trial evaluating an IO-IO combination; the experimental arm received nivolumab 3 mg/kg combined with ipilimumab 1 mg/kg every 3 weeks for 4 doses, followed by nivolumab 3 mg/kg every 2 weeks, compared with sunitinib 50 mg daily for 4 weeks then 2 weeks off. Random assignment was stratified by region and IMDC risk category in previously untreated advanced ccRCC patients. The primary end points were ORR, PFS, and OS. (ClinicalTrials.gov identifier: NCT02231749). 

The **KEYNOTE-426** evaluated in the experimental arm pembrolizumab 200 mg every 3 weeks with axitinib 5 mg twice daily versus sunitinib. Random assignment was stratified by region and IMDC risk category in previously untreated advanced ccRCC patients (ClinicalTrials.gov identifier: NCT02853331).

The **CLEAR** evaluated in the experimental arm pembrolizumab 200 mg every 3 weeks with lenvatinib 20 mg daily versus sunitinib. Random assignment was stratified by region and MSKCC risk category in previously untreated advanced ccRCC patients (ClinicalTrials.gov identifier: NCT02811861).

The **Checkmate 9ER** evaluated in the experimental arm nivolumab 240 mg every 2 weeks plus cabozantinib 40 mg daily or sunitinib. Random assignment was stratified by region, IMDC risk category, and tumor expression of PD-L1 in previously untreated advanced ccRCC patients (ClinicalTrials.gov identifier: NCT03141177).

Even if the IMDC risk score was initially developed during the VEGFR-TKI era, its use continues in the era of frontline combination immunotherapy. The IMDC risk score is determined by evaluating six risk factors: time from initial diagnosis to randomization of less than 1 year, Karnofsky performance status score of less than 80, hemoglobin level below the lower limit of the normal range, corrected serum calcium level above the upper limit of the normal range, absolute neutrophil count above the upper limit of the normal range, and platelet count above the upper limit of the normal range. Patients belonging to the favorable-risk group have an IMDC score of 0, intermediate risk corresponds to a score of 1 or 2, and poor risk corresponds to a score of 3 to 6 [21]. To this day, the IMDC score is the only score prospectively validated in multiple trials, and it is thus used by clinicians to assess disease aggressiveness and choose a therapeutic path.

These trials led to the four combination therapies currently approved by the FDA and EMA in the first-line setting, supported by an advantage in OS over sunitinib in phase III clinical trials: axitinib plus pembrolizumab, cabozantinib plus nivolumab, lenvatinib plus pembrolizumab, and nivolumab plus ipilimumab. These combinations have the most evidence for poor/intermediate IMDC risk groups, and nivolumab plus ipilimumab has been approved by the FDA and EMA only for this group of patients. However, there are no trials of head-to-head comparisons for these different treatments. Other treatments, such as avelumab plus axitinib and atezolizumab plus bevacizumab, have been evaluated in phase III trials, showing a benefit for progression-free survival, while the data are still immature for the avelumab/axitinib combination [22], whereas the phase III trial final analysis of atezolizumab plus bevacizumab showed a lack of improvement on overall survival [23]. Another phase III trial, the COSMIC-313, has evaluated, for the first time, a triplet therapy, with nivolumab, ipilimumab, and cabozantinib compared with nivolumab plus ipilimumab, in the first-line setting in patients with mRCC intermediate or poor IMDC risk groups. Data gathering for OS is ongoing, with a current benefit in PFS for the experimental group (95% CI, 14.0 months—not yet estimated vs. 7.7 months to 18.2 months), yet the benefit is evident only within the intermediate-risk group at a pre-specified subgroup analysis [24]. Another phase III trial, RENOTORCH, has recently compared sunitinib versus axitinib in combination with another PD-1 inhibitor, toripalimab, showing a benefit in PFS and a trend in improved OS at an early follow-up [25].

Hereafter, we summarize the evidence from the four phase III clinical trials that demonstrated an OS benefit compared to sunitinib in the first-line setting for advanced ccRCC, from 1 January 2017 to 1 June 2023: **CheckMate 214** (nivolumab plus ipilimumab), **KEYNOTE-426** (pembrolizumab plus axitinib), **CheckMate 9ER** (nivolumab plus Cabozantinib), and **CLEAR** (lenvatinib plus pembrolizumab).

**Table 1:** Characteristics of patients, response to treatment, therapy discontinuation, and subsequent therapies in registration-directed trials that demonstrated a benefit in OS for mRCC.

	CheckMate 214: Nivolumab + Ipilimumab [26]	KEYNOTE-426: Pembrolizumab + Axitinib [27,28,29,30]	CheckMate 9ER: Nivolumab + Cabozantinib [31]	CLEAR: Lenvatinib + Pembrolizumab [32,33]
**POPULATION AND FOLLOW-UP**				
Median follow-up for OS	67.7 months	30.6 months ^¤^	32.9 months	33.7 months †
Number of patients in the ITT population in the experimental arm versus sunitinib arm (safety population)	550 (547) versus 546 (535)	432 (429) versus 429 (425)	323 (320) versus 328 (320)	355 (352) versus 357 (340)
Pts characteristics according to IMDC score: Favourable/Intermediate/Poor (% *) ^	125 (22.7)/ 334 (60.7)/ 91 (16.5)	138 (31.9)/ 238 (55.1)/ 56 (12.7)	74 (22.9%)/ 188 (58.2%)/ 61 (18.9%)	110 (31.0)/ 210 (59.2)/ 33 (9.3)/ 2 pts could not be evaluated (0.6)
Number of patients that discontinued therapy at data cut-off (% *)	516 (93.8)	312 (72.2)	228 (70.6)	238 (67.7)
**OUTCOMES**				
Discontinuation of one or both experimental drugs for drug-related AEs ^#^	148 (27.0). (148 discontinued both drugs; single drug interruption was not possible)	148 (34.5). (92 discontinued at least pembrolizumab, 56 axitinib, 28 both)	87 (27.2). (34 discontinued nivolumab only, 29 dc cabozantinib only, 20 both)	131 (37.2) reported as any Aes leading to discontinuation (90 discontinued at least lenvatinib, 101 pembrolizumab, 47 both drugs) †
Discontinuation of one or both experimental drugs for AEs unrelated to treatment (% ^#^)	40 (7.3)	Not reported	24 (7.4)	See above. †
Discontinuation for disease progression (% ^#^)	266 (48.6)	181 (42.2)	129 (40.3)	116 (33.0) †
Completion of ICI and continuing TKI where applicable (% ^#^)	Not applicable	19 (4.4)	Not reported.	101 (28.9)
Number of patients that had the best overall response to experimental drug: CR/PR/SD/PD/not evaluated (% *)	64 (11.6) 152 (27.6) 198 (36.0) 97 (17.6) 39 (7.1%)	38 (8.8)/CR increased to 10% over time ^§^ 222 (51.4) 100 (23.1) 49 (11.3) 23 (5.3)	40 (12.4) 140 (43.3) 105 (32.5) 20 (6.2) 18 (5.6)	61 (17.2) 191 (53.8) 68 (19.2) 19 (5.4) 16 (4.5)
Number of patients that had the best overall response to sunitinib: CR/PR/SD/PD/not evaluated or unable to determine (% *)	14 (2.6) 163 (29.9) 230 (42.1) 77 (14.1) 62 (11.3)	13 (3.0) 158 (36.8) 150 (35.0) 74 (17.2) 34 (7.9)	17 (5.2) 76 (23.2) 134 (40.9) 45 (13.7) 56 (17.1)	15 (4.2) 114 (31.9) 136 (38.1) 50 (14.0) 42 (11.8)
Median duration of response (DoR) in the experimental treatment group versus the sunitinib group (95% CI)	Not reached (59.0–not estimable) versus 24.8 months (19.7–30.1)	23.5 months (19.4–29.0) versus 15.9 months (13.8–20.4)	23.1 months (20.2–27.9) versus 15·1 months (9.9–20.5)	26.0 (22.2–41.4) versus 14.7 (9.4–16.8)
Ongoing responses (as % of responders; as % of ITT population) at median follow-up time reported in the first row	136 (47.7; 24.9)	62 (23.9; 14.4) ^§^	88 (48.9; 27.2)	Not reported
**SAFETY**				
Any G3-4 AEs (exp arm versus sunitinib arm) (% ^#^)	373 (68.2) versus 417 (77.9)	Not reported as all G3-4 AEs	Not reported	74.1% versus 60.3% †
Treatment-related G3-4 AEs (exp arm versus sunitinib arm) (% ^#^)	263 (48.1) versus 344 (64.3)	283 (66.0) versus 259 (61.0)	208 (65.0) vs. 172 (53.8)	252 (71.6) versus 200 (58.8) (includes only trAEs occurring in 10% or more of the patients) †
Treatment-related deaths (exp arm versus sunitinib arm) (% ^#^)	8 (1.5) versus 5 (0.9)	4 (0.9) versus 6 (1.4)	1 (0.3) versus 3 (0.9)	4 (1.1) versus 1 (0.3) †
Any death not related to PD or treatment (exp arm versus sunitinib arm) (% ^#^)	Not reported	19 (4.4) versus 17 (4.0)	3 (0.9 vs. 3 (0.9)	Not reported
**SUBSEQUENT THERAPIES**				
Number of patients that received subsequent systemic therapies after discontinuation for any reason (as % of patients that discontinued treatment ^#^)	305 (55.7)	204 (47.2) ^§^	70 (30.7)	132 (37.5)
Type of subsequent systemic therapies after discontinuation (total number of patients) ~	Antiangiogenic 165% (504) PD-(L)1 inhibitor 24.3% (74) MTOR inhibitor 20.3% (62) CTLA-4 inhibitor 1.6% (5) Other 0	Antiangiogenic 88.2% (180) PD-(L)1 inhibitor 21.6% (44) Everolimus 26% (53) CTLA-4 inhibitor 3.9% (8) Other 7.4% (15) ^§^	Antiangiogenic 87.1% (61) PD-(L)1 inhibitor 22.9% (16) MTOR inhibitor 11.4% (8) CTLA-4 inhibitor 10% (7) Other 8.6% (6)	Antiangiogenic 92.4% (122) PD-(L)1 inhibitor 27.2% (36) MTOR inhibitor 6.8% (9) CTLA-4 inhibitor 4.5% (6) Other 12.1% (16)

All the data refer to the experimental arms when not otherwise specified. AEs: Adverse events. ^¤^ An updated follow-up regarding OS for The KEYNOTE-426 has been published, providing an extended 5-year comparison for OS, which falls beyond the scope of this table. Additionally, this update encompasses the duration of treatment; however, it lacks a confidence interval. It is noteworthy to mention that the mean values for DoR differ only by a few weeks [34]. * As % of the intention to treat (ITT) population. ^#^ As % of the safety population. ^ In the CLEAR trial, the IMDC score was not used for stratification; the Memorial Sloan Kettering Cancer Center (MSKCC) score was used instead. ^§^ Data are obtained from the 42.8-month follow-up papers [28,29,30]. † The updated study for the CLEAR trial with the data cut-off on 31 March 2021, with a median of 33.7-month follow-up for OS did not provide any safety analysis [32]; therefore, all safety analysis for the CLEAR trial refers to the data with the cut-off date on 28 August 2020 (26.6-month median follow-up for overall survival) [33], aside from ≥G3 AEs, updated at a 4-year follow-up [35]. ~ Patients may have received more than one line of therapy. All trials have used Recist 1.1 criteria to assess radiological response as a primary outcome.

## 3. Possible Outcomes in mccRCC following a Combination Treatment

Patients receiving ICI-based combination therapies may experience different types of responses due to the nature of these therapies. Disease progression may happen during treatment, as oligo or systemic progression; patients treated with ICI doublet or monotherapy, in addition, may experience a pseudoprogression, defined as a transient increase in size or detection of previously undetectable lesions, followed by a response or stability in subsequent imaging. 

Discontinuation of first-line ICI-based combination treatment can occur after treatment-related adverse events, completion of ICI treatment per study protocol, for disease progression while on treatment, or for maximum clinical benefit or complete response (graphical abstract).

The main reason for treatment discontinuation across all the pivotal clinical trials was disease progression, followed by drug toxicity. Notably, all trials excluded patients with active brain metastases, for which fewer data are available.

**Figure 2:**
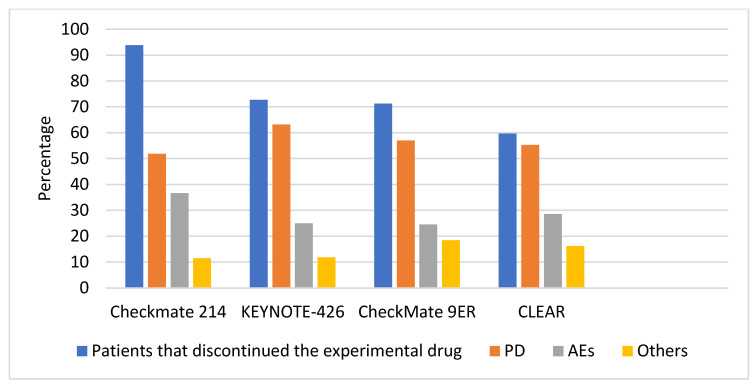
Percentage breakdown of patients discontinuing therapy within the safety population (depicted in blue) and highlighting the causes leading to drug discontinuation as a proportion of the total patients who discontinued treatment (represented by different colors) (ref. Figure 1).

In Table 1, we presented the efficacy, safety outcomes, and subsequent treatments observed in the four phase III clinical trials showcasing an OS benefit over sunitinib in the first-line treatment of advanced ccRCC. These results were published between 1 January 2017, and 1 June 2023. Meanwhile, Table 2 comprises our assessment of the same trials, focusing on reasons for the discontinuation of study drugs, referencing the CONSORT diagrams. This evaluation was necessary as the data provided in the diagrams differed slightly from the information presented in the main text of the respective papers, offering additional insights and information. Figure 2 provides a visual representation of the breakdown of patients discontinuing therapy within the safety population, visually represented in blue. The figure emphasizes various causes leading to drug discontinuation, each depicted by different colors, showcasing their respective proportions concerning the total number of patients who discontinued treatment. The increase in patients discontinuing the drug across different trials is mostly related to a different duration of follow up.

**Figure 3:**
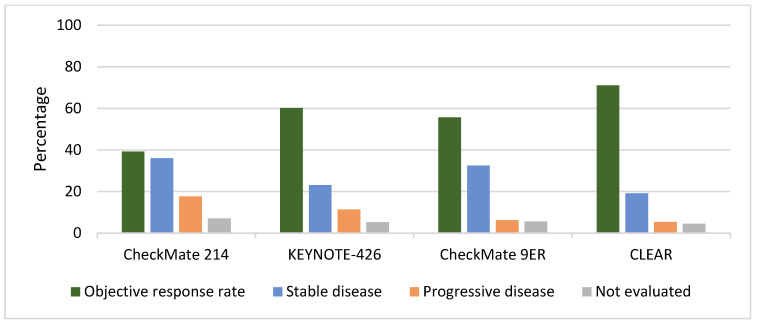
Distribution of best overall responses (bOR) across trials shown in Table 1.

In Figure 3, we show the different responses across the aforementioned trials. This analysis should not be interpreted as a direct comparison, considering the heterogeneity of the trials, the different distribution of IMDC patients, the stratification for MSKCC in the CLEAR trial, and the different duration of responses, but it may be used to evaluate the overall outcomes from single treatments across the different trial populations. For direct comparisons across trials, there is an essential need for perspective evidence. Presently, the selection of treatment relies heavily on the expertise of clinicians and an assessment of anticipated drug toxicities.

### 3.1. Progression While on Treatment

Primary progressive disease occurred in 17.6% of patients in the CheckMate 214 trial, 11.3% of patients in KEYNOTE 426, 6.2% of patients in Checkmate 9ER, and 5.4% of patients in CLEAR; discontinuation for progressive disease occurred, respectively, in 48.6%, 42.2%, 40.3% and 33% of patients. While the aim of choosing a first-line treatment in mRCC is beyond the scope of this review, the elevated incidence of primary progressive patients for the ICI-ICI combination reported in Figure 3 should prompt physicians to consider the potential benefits and risks associated with this approach. Direct comparisons across treatments or biomarkers for better treatment guidance are awaited. Currently, factors to weigh beyond IMDC risk groups could include the presence of multiorgan metastasis, bone or liver involvement, symptomatic brain metastasis, physician expertise, comorbidities, and expected treatment toxicities. The use of PD-L1 expression may have a role as well [6,36,37,38]. The vastly superior outcomes in sarcomatoid RCC compared to sunitinib at a 5-year follow-up warrant the use of ICI-ICI in most patients in this sub-population while awaiting the long-term outcomes from other combinations [39]. Disease progression eventually led to discontinuation in the majority of patients across all trials, ranging from 51.9% in the CheckMate 214 to 63.1% in the KEYNOTE 426 (Figure 2).

#### 3.1.1. Pseudoprogression

Patterns of response and progression to immunotherapy may differ from those observed with drugs such as chemotherapy and molecularly targeted therapies. One important difference is that ICIs may initially cause a transient increase in the size of tumors or the detection of previously undetectable lesions, followed by a response or stability on subsequent imaging. This phenomenon, known as pseudoprogression (PP), was first reported in patients with melanoma who were treated with ipilimumab and is likely due to immune infiltration of the malignant lesions [40,41]. The rate of pseudoprogression has usually not exceeded 10% of patients across tumor types [42]. Since the radiographic appearance cannot be differentiated from that of a true progression, the clinician must be aware of the possibility of a PP, as its misrecognition can lead to worse outcomes for patients if ICI treatment is discontinued.

Differentiating PP from a true progression can be hard for the clinician and the radiologist, as at the present time, there are no biological or radiological markers of true progression beyond performing a biopsy showing immune infiltration or necrosis [41]. To guide clinicians and standardize care, in an effort to create a “common language” for immune-related response [43], several immune response criteria have been created through the years. Wolchok et al., in 2009, created the immune-related response criteria (irRC) to assess radiological tumor response to immune checkpoint inhibitors based on WHO criteria [44]. In 2013, Nishino et al. proposed a simplified version of irRC using a unidimensional model (irRECIST). In 2017, the RECIST working group produced a consensus based on a re-evaluation of RECIST 1.1 (iRECIST) [45]. All these frameworks, in the case of progression, require, when feasible, a subsequent scan at ≥4 weeks to confirm progression or to disprove it; the main difference is the incorporation of the measurements of new lesion(s) in the tumor burden, which is not added in the iRECIST criteria, conversely to the others [46].

PP has been poorly studied in clinical trials of mRCC patients. However, the incidence of PP has been well documented in various cancer types in the context of ICI monotherapy. A systematic review and meta-analysis by Park et al. [42] of data from 3402 patients treated with ICIs found an incidence of PP ranging from 4.5% to 8.0%, depending on the definition and type of cancer. In melanoma, the cancer type with the longest experience with ICI treatment, PP has been reported at a rate between 6% and 12% [47].

The most relevant data for mRCC come from a systematic review from 2018 by Soria et al. [48], who analyzed data from two phase I, one phase II, and one phase III clinical trial(s) of nivolumab in patients with pre-treated mRCC. The review found that 9% of 34 patients in a phase I trial, 15% of 168 patients in a phase II trial, and 13% of 153 patients treated beyond progression in the phase III CheckMate 025 trial experienced PP. A subgroup analysis of the CheckMate 025 revealed a difference in overall survival between patients who received ICI treatment beyond progression and those who did not (median overall survival of 20.4 (95% CI: 17.3–not reached) versus 12.2 months (95% CI: 9.5–14.6), respectively). However, statistical significance was not assessed, as the primary analysis of the study did not encompass formal statistical testing or direct comparative assessments for this specific outcome. The decision to continue treatment beyond progression was based on clinical factors such as good Karnofsky performance status at baseline, shorter time to response, lower incidence of new bone lesions, improved quality of life, and less deterioration in Karnofsky performance status during treatment [49]. A subsequent systematic review did not identify any additional prospective trials on PP in kidney cancer [42]. PP has also been documented in other cancers for the combination of nivolumab plus ipilimumab [50] or ipilimumab alone [51], and it was first reported in patients receiving ipilimumab monotherapy for melanoma [52].

In a pooled analysis by Manitz et al. [53] using data from 1765 patients with 12 types of advanced solid tumors treated with avelumab, including 82 RCC cases, they found that irRECIST and RECIST 1.1 were discordant in 8.3% of cases (6.1% of RCC), underpinning possible pseudoprogressions. Of the discordant patients, 91.8% had an irRECIST SD and a RECIST 1.1 progression (135/147). In an effort to characterize the patients in the discordant subgroup that could benefit from continued treatment, Manitz et al. found that the type and amount of progression can help evaluate PP versus true progression, as the incidence of PD as defined by all RECIST 1.1 criteria (target, non-target, and new lesions) was higher in the concordant disease progression subgroup (11.3%) compared to the discordant subgroup (1.4%); moreover, the discordant subgroup had more often a RECIST 1.1 PD based on a single criterion (55.1% versus 28.9%). Interestingly, most of the patients in the discordant subgroup appeared to benefit from continued treatment, with the survival curve for the discordant subgroup falling between the PD and SD/PR concordant subgroups (95% CI: 5.8 to 10.0 months vs. 3.8 to 4.8 months vs. 17.2 to 21.5 months).

The time from treatment initiation to progression is likely an important factor to consider when assessing the probability of PP, considering that the majority of PP cases, observed across a diverse spectrum of cancer types, typically occur within 6 months after the initiation of treatment. It is noteworthy that subsequent responses may take several months to occur [54,55,56].

The role of PP in ICI-TKI combinations is not well understood, as there are limited data available on this topic. In a phase II clinical trial evaluating lenvatinib plus pembrolizumab in subsequent lines of therapy, there was no difference in PFS and ORR with either irRECIST or RECIST 1.1 criteria. The PFS were 11.7 and 11.3, respectively, and the ORR were 54.8% and 51.9%, respectively. However, all patients had already progressed on a prior line of therapy to a PD-(L)1 inhibitor, making the results hardly applicable in evaluating PP in the first-line setting [57].

All the trials in Table 1 formally used RECIST criteria to assess radiological response as a primary outcome, but in every trial, treatment beyond progression was allowed, and the JAVELIN Renal 101 and Keynote-426 trials solicited to continue treatment and re-evaluate progressive disease at 4 weeks when feasible, depending on clinical benefit and tolerance of the drug. The CLEAR trial protocol initially used irRECIST criteria to report on exploratory endpoints, but they were removed from the updated protocol.

To our knowledge, no data have been published regarding the subgroups of patients treated beyond progression or for pseudoprogressive disease in the JAVELIN Renal 101, nor in the CLEAR trials. Some data have been reported for the CheckMate 214 and the Keynote 426 trials; these will be evaluated in Section 3.1.6.

Given the current knowledge, PP may occur in mRCC treated with ICI, but most data come from patients treated with monotherapies; thus, physicians should be careful in the setting of IO-TKI combinations where PP has not been properly characterized.

#### 3.1.2. Hyperprogressive Disease

Hyperprogressive disease (HPD) has been defined as a distinct entity from disease progression, defined as an acceleration of disease growth after the start of a new cancer treatment. HPD has been reported in several cancer histologies with different cancer treatments; most of the evidence has focused on ICIs, but it has first been reported in phase I trials before the advent of immunotherapies [58] and later in chemotherapy regimens and other agents [59,60]. Currently, there is no universally accepted clinical, radiological, or biochemical definition of HPD [61,62]. 

A systematic review evaluating the different definitions of HPD found that it was divided into four categories: tumor growth rate ratio (TGR), tumor growth kinetics ratio, early tumor burden increase, and combinations of these categories. The use of any of these different definitions led to over- or underestimation of HPD according to the others criteria, and each definition was not unanimous, with different possible equations, such as for TGR [58,62,63]. Many studies reporting on HPD did not evaluate pre-treatment growth parameters [64,65], thus evaluating the speed of progression rather than an acceleration with respect to pre-treatment growth parameters. Gandara et al. have proposed categorizing patients with a marked progression at the first assessment as having a “fast progression” [66].

A correct assessment would have to consider the natural cancer kinetic, represented by an exponential equation when not hampered, and evaluate whether it changes course during treatment, which may or may not be due to treatment itself, such as that of Gomez et al. [58].

The first report of HPD in ICI-treated patients by Champiat et al. [67], using the different definition of TGR first proposed by Ferté et al. [63], retrospectively analyzed 131 patients treated with anti-PD (L)1 inhibitors in monotherapy, finding HPD, according to their definition, in 9% of patients; in this group of patients, they also found a worse overall survival. The group included only nine renal cancer patients, none of whom experienced HPD. HPD was more common in other cancer types, although the size of these patient groups was small (in some cases as small as two patients). 

Prospective cohorts have yet to demonstrate whether HPD represents a distinct pattern of disease progression from rapid progression [68]. However, some researchers argue that HPD may simply reflect the natural disease course in patients in which ICIs lack activity. Kang et al. analyzed data from two phase III trials of nivolumab or nivolumab plus ipilimumab in small-cell lung cancer and nivolumab alone in gastric or gastroesophageal junction cancer and found no evidence of HPD in either the overall analysis or the non-responder population compared to the placebo cohorts [68]. Data showing the occurrence of HPD with other treatments may support the lack of a specific pattern due to immunotherapy [58,59,60]. Currently, many hypotheses have been made on the possible mechanism of HPD [69], but no pathological mechanism has been confirmed in patients. 

In a retrospective analysis by Hwang et al. [70] of 102 patients with RCC treated with PD(L)1 as monotherapy or in combination with targeted agents or CTLA-4 inhibitors, HPD, defined as a time to treatment failure <2 months, >50% increase in the sum of the longest diameter of the target lesions, and >2-fold increase in TGR according to the formula of Ferté et al. [63], occurred in a negligible percentage of patients with RCC (0.9%), whereas patients with urothelial carcinoma (UC) from the same analysis had much higher HPD rates (11.9%). In the study from Hwang et al., HPD was defined as time to treatment failure <2 months, a >50% increase in the sum of the longest diameter of the target lesions, and a >2-fold increase in tumor growth rate. As would be expected, having an HPD as the best response was found to be negatively associated with prognosis, with a median OS lower than that of the patients with PD as the best response in the UC cohort (3.5 months (95% CI, 2.3–4.7) versus 6.4 months (95% CI, 3.8–9.0 months)).

Based on the available literature presented, the incidence of HPD in kidney cancer is likely to be low with ICI monotherapy or ICI-ICI combination and may not occur at all with ICI-TKI combinations, even when accounting for different definitions. The occurrence of HPD, whereas controversial, would rarely, if ever, underpin a PP, according to the presentation of PP from the data from Manitz et al. [53]; therefore, the clinician, when presented with swift progressions, should quickly consider other treatment options.

#### 3.1.3. True Progression

True progression is the most common outcome after first-line therapy and can be subcategorized into oligo or systemic progression.

#### 3.1.4. Oligoprogression

A cancer may be evaluated as oligoprogressive while ongoing a systemic treatment, if the patient experiences a growth in a few metastatic sites while enduring a sustained response in others. Oligoprogression (oligoPD) may be due to local differences in the tumor microenvironment or to cancer heterogeneity, conferring resistance to the ongoing therapy. There is no clear consensus on what represents an oligoprogression. Several studies evaluating radiotherapy and oligoprogressive disease use documented progression in up to 3 or 5 individual lesions, confirmed in consecutive imaging 2–3 months apart, as criteria [71,72,73].

There is a paucity of data on the prevalence of oligoprogression in mRCC. In the CheckMate 214 trial, through an analysis of the patterns of progression at the timepoint in which 299/550 (54.4%) patients had progressed, ref. [74] showed that in the Nivolumab + Ipilimumab arm, 35.5% of the progressions resulted from new lesions only (NL), and in patients with an initial confirmed response, most NL progressions occurred in a single organ (34/36 (94.4%)); therefore, at the time of analysis, at least 11% of all patients that had progressed had an oligoPD. No data regarding oligoPD are available for the other trials evaluated.

If oligoPD occurs, the clinician has several options to consider, including using local therapy to eliminate resistant clones while continuing the same systemic therapy, continuing therapy beyond progression, switching to a different therapy, or discontinuing systemic treatment altogether. Focal therapies can prolong the effectiveness of ongoing treatments and conserve limited subsequent therapeutic options in the treatment of oligoprogressive mRCC. In addition, targeting resistant metastases with a focal approach may also improve survival outcomes.

The most common local therapy approach in oligoPD mRCC involves using local radiotherapy treatments (such as stereotactic body radiation therapy (SBRT), also called stereotactic ablative radiation (SAbR), or stereotactic radiosurgery (SRS)). 

Radiation therapy has been proposed to enhance the activity of immune-modulating agents through various mechanisms, including priming the immune system, attracting immune cells to the tumor microenvironment, and altering the immunosuppressive effects of the tumor microenvironment to promote a more pro-immunogenic microenvironment [75,76]. Current clinical evidence depicts a complex scenario, as a phase II trial evaluating the combination of concomitant nivolumab and SBRT in mRCC did not demonstrate improved outcomes compared to historical data [77], nor did a phase II randomized trial in head and neck squamous cell carcinoma that showed no benefit of adding concomitant SBRT to nivolumab alone [78]. This suggests that adding radiotherapy at the same time as immunotherapy may lead to worse outcomes than starting radiotherapy before immunotherapy when feasible, but the findings may not be applicable to oligoprogressive disease. Adjusting the timing of radiotherapy and ICI combinations appears to offer greater efficacy,, such as the evidence from a randomized trial of radiotherapy followed by pembrolizumab in non-small-cell lung cancer that showed a doubling of ORR and OS in the group receiving radiotherapy one week before pembrolizumab [79], and another small trial evaluating a single-arm trial treating patients with oligometastatic mRCC with SAbR on all lesions followed by eight courses of pembrolizumab, which achieved a PFS of 74% at two years in patients in third-line therapy or subsequent lines of therapy [80]. 

Regarding oligoprogression in mRCC, most of the evidence supporting the use of SAbR for oligoPD is retrospective [81,82,83,84,85,86,87,88], except for two prospective small phase II clinical trials investigating the role of therapy SAbR for oligoPD in mRCC, which show a prolongation of ongoing therapies and good local control. One trial involved oligoprogressive (≤5) mRCC treated with sunitinib or pazopanib, showing a median PFS of 9.6 months (95% CI: 7.4–20.5) and a local control at 2 years of 96% after stereotactic radiotherapy [89]. The other prospective trial in oligoprogressive (≤3) mRCC included patients treated with targeted therapy, ICIs, or a combination of ICIs and TKIs, showed a median PFS of 11.1 months (95% CI: 4.5–19.3) after stereotactic ablative radiotherapy, with 100% local control at 10.4 months of median follow-up, and no grade 4 or 5 toxicities. Both trials excluded patients with intermediate or poor risk according to the IMDC criteria [90].

While a review of the available knowledge about local therapies for oligoprogression is beyond the scope of this article, we consider local therapies to be increasingly supported by the literature and becoming widespread practice for managing oligoprogressive disease since the introduction of targeted inhibitors [81,82]. Significant questions need yet to be addressed, including determining optimal dose/fractionation schemes for radiotherapy, selecting the appropriate timing for treatment, and identifying the patients who may benefit from these therapies. More data will come from two phase II clinical trials currently undergoing: NCT04974671, a phase II trial evaluating radiotherapy to oligoprogressive (≤5) lesions in mRCC treated with any ICI-containing regimen prior to trial enrolment, and NCT04299646 (GETUG-StORM-01), a phase II trial evaluating radiotherapy to oligoprogressive (≤3) mRCC after first- or second-line treatment [72,73].

#### 3.1.5. Systemic Progression

The scenario of systemic progression is the most common, compelling the physician to consider a change of therapy, discontinue oncological treatments, and pursue palliative supportive care or continue the same therapy beyond progression if the patient is still experiencing a clinical benefit. 

#### 3.1.6. Therapy beyond Progression

In all first-line ICI-based combination phase III trials listed in Table 1, protocols allowed the continuation of treatment beyond progression (TBP) for clinical benefit.

In the first analysis of the CheckMate 214, 29% of patients in the experimental arm (157/550) were treated beyond progression [91], with a median duration of treatment of 3.88 months beyond progression (interquartile range IQR: 1.38–10.48 months); the third quartile reached 10.48 months of time on TBP, possibly underpinning some pseudoprogressions. For the sunitinib arm, the median time on TBP was 1.22 months (IQR 0.53–3.98 months), conferring a smaller benefit. At the extended follow-up analysis [92], the swimmer plots evaluating patients experiencing a PR/CR and a subsequent PD show an ongoing TBP in the experimental arm of six patients, with four being on therapy for more than 48 months and one in the sunitinib arm with an ongoing treatment of 20 months.

For the Keynote 426, a report from the Japanese cohort on the swimmer plot analysis shows that a part of the patients treated beyond progression continued treatment for more than a year [93].

No data for TBP outcomes are available for the CheckMate 9ER and CLEAR trials.

#### 3.1.7. Changing Systemic Therapy

As the most common outcome for patients in the trials in Table 1 was discontinuation of therapy for disease progression, evaluating the second-line therapy is of outmost importance. The average number of patients who did not undergo a successive line of therapy after treatment discontinuation is slightly above 40% for each trial, representing an even more critical hurdle for real-world practice, as patients are less selected than those in clinical trials.

Regardless of the combination used in the first-line treatment, almost all patients that underwent subsequent therapies at discontinuation eventually received an antiangiogenic therapy; 22% to 27% of patients received therapy with a PD-(L)1 inhibitor, and mTOR inhibitors were provided for 7% to 26% of patients. Ipilimumab monotherapy was infrequently administered as a subsequent line of treatment, with the highest use in the CheckMate 9ER (10%). There is a lack of data on outcomes from these subsequent therapies.

Progression-free survival 2 (PFS2), defined as the time from randomization to progression on the first subsequent therapy, has been reported for the KEYNOTE-426 and the CLEAR trial, without subgroup analysis for single drugs.

PFS2 from all treatments for the KEYNOTE-426 trial [30] showed an aggregate improvement in PFS2 from the first subsequent therapy in the experimental arm versus sunitinib (40.1 months versus 27.7 months). No data were reported for the median duration of subsequent therapies.

In the CLEAR study [94], PFS2 was longer in the experimental arm than in the sunitinib arm (median not reached versus 28.7 months; HR, 0.50; 95% CI 0.39–0.65), and the median duration of the first subsequent anticancer therapy was slightly shorter, even if not statistically significant, in the experimental arm (5.2 months, range 0.10–30.23) compared to that in the sunitinib arm (6.8 months, range 0.03–30.72).

Data from the AFTER-IO retrospective study [95] involving patients enrolled in the CheckMate 025 or CheckMate 214 trials in Japanese centers who underwent a subsequent treatment after Nivolumab or Nivolumab + Ipilimumab discontinuation reported the effectiveness of sunitinib and axitinib, with a high disease control rate (DCR), achieving, respectively, 100% and 78%, with similar median PFS2 of 32.0 versus 26.1 months and a median OS not reached for both groups, at a 48 months follow-up. The patients had previously discontinued treatment for progression (67%) or adverse events (33%). 

The data from the JAVELIN Renal 101 trial [96] reported a better PFS2 in the experimental arm. No trials reported PFS2 for single therapies.

Only one phase III randomized study, the CONTACT-03 trial, has been performed so far in this setting; it is a phase III trial in mRCC after progression to prior ICI treatment in the second- or third-line settings, in which atezolizumab plus cabozantinib vs. cabozantinib alone were administered to patients. The results of the trial do not support the addition of ICI to previously treated patients, as there was no PFS or OS benefit for the combination against cabozantinib alone (PFS 95% CI 9.8–12.3 months with atezolizumab–cabozantinib and 10.0–12.5 months with cabozantinib alone) [97].

A systematic review evaluated four perspective phase II clinical trials (OMNIVORE, FRACTION RCC, HCRN GU16-260, TITAN-RCC) [98,99,100,101] to assess the effectiveness of ipilimumab in mRCC after nivolumab SD or PD, of which only TITAN-RCC and FRACTION-RCC included patients exposed to prior antiangiogenic treatment, with a pooled ORR of 10% [102].

In another phase II trial of the FRACTION platform, 46 patients with mRCC who had progressed on previous immuno-oncology combination therapy were treated with nivolumab and ipilimumab upfront. Median follow-up was 33.8 months, and the ORR was 17.4% (they were all partial responses). A total of 41.3% of patients achieved stable disease, while 30.4% had progressive disease. The median DoR was 16.4 months (range 2.1+ to 27.0+ months) [99].

A single-arm phase II clinical trial evaluated lenvatinib plus pembrolizumab in 104 patients having a PD during or following an ICI, of which 65% had prior IO-TKI therapy and 37% had prior IO-IO treatment. The PFS was 11.3 months in the ITT, and the ORR was 58.8% in the IO-TKI group and 47.4% in the IO-IO group, showing the efficacy of the combination in subsequent lines of the therapy [57]. In a retrospective cohort by Dizman et al. [103], patients treated in second-line or more with pembrolizumab plus axitinib had an ORR (all PR) and DCR of 25.0% and 66.6%, respectively, with a median DoR of 10.6 months and a PFS of 9 months. Notably, the cohort of patients treated in the second-line setting following nivolumab–ipilimumab had an ORR of 31.4% and a median PFS of 11.1 months, showing improved results in regard to the other treatment sequences examined, warranting future investigations. Whether these combinations lead to superior outcomes against single-agent TKI remains to be evaluated, but current evidence from the CONTACT-03 trial is discouraging. Single-target therapies in a retrospective cohort by Auvray et al., evaluating TT in the second-line setting after nivolumab–ipilimumab, showed a similar PFS with a median of 8 months and an ORR of 36% [104].

### 3.2. Discontinuation for Adverse Events

Adverse events (AEs) are a common occurrence during treatment, and they can significantly impact patients’ quality of life and may impact survival outcomes if severe. Proper management of AEs is essential for optimizing treatment and ensuring the safety and well-being of patients. Accurate identification of the responsible agent is crucial for managing AEs that occur in combination with different treatments.

AEs were the second most common reason for discontinuation in the pivotal trials. Immune-related adverse events (irAEs) pose diagnostic challenges in the differential diagnosis of non-immune related AEs but require prompt and targeted care. The criteria for discontinuation for immune-related adverse events (irAEs) have been changing in recent years, with guidelines relying on expert consensus due to a lack of strong prospective data. Recent guidelines suggest that most grade 3 and most grade 4 irAEs should permanently discontinue ICI therapy, except for endocrinopathies [105].

In the CheckMate 214, 27% of patients discontinued the study drug due to irAEs, but 64% experienced a toxicity of G3 or more, and 7% discontinued for toxicities reported as unrelated to treatment [18]. The OS for patients who discontinued treatment for trAEs was equivalent to that of the patients who did not (95% CI 48.1–NE versus 49.8–NE) [106]. In a previous report from the CheckMate 214, it was found that among patients experiencing discontinuation for irAEs, almost half (42%) were treatment-free at 24 months, even if 47% of the discontinuation occurred during the induction (N+I) phase [107].

The outcomes for patients discontinuing therapy for AEs for the remaining trials have not been reported.

In the KEYNOTE-426, 45.9% of patients in the experimental arm discontinued one or both study drugs for toxicity, of which 34.3% were for trAEs.

In the CheckMate, 9ER TRAEs led to discontinuation of either study drug in 25.7% of patients in the experimental arm (11% discontinued nivolumab only, 9% discontinued cabozantinib only, 7.5% discontinued both nivolumab and cabozantinib sequentially or simultaneously), and 7.4% discontinued for toxicities reported as unrelated to treatment.

In the CLEAR trial at the first interim analysis, TRAEs of any grade led to discontinuation in 40.6% of patients (lenvatinib 25.6%; pembrolizumab 28.7%; both drugs 13.4%).

A systematic review by Zhao et al. evaluated 789 ICI rechallenges from retrospective studies, finding a pooled all-grade irAEs incidence at rechallenge of 34.2% (OR, 3.81; 95% CI, 2.15–6.74), but high-grade irAEs incidence was similar to initial ICI treatment at 11.7% [108]. In an observational, cross-sectional, and pharmacovigilance study of safety reports from the World Health Organization database VigiBase, 24,079 irAE cases were evaluated and rechallenging with the same ICI resulted in a 28.8% recurrence rate of the same irAE. Colitis, hepatitis, and pneumonitis had higher recurrence rates compared to other irAEs, while adrenal events had a lower recurrence rate [109]. Some authors advocate for prophylactic selective immunosuppressive treatment, such as anti-IL-6 monoclonal antibodies or α_4_β_7_ inhibitors, for ICI resumption after severe irAEs in patients without other effective treatment options [110].

The efficacy data are promising even if few are available for mRCC. A cohort of patients with mRCC treated with at least two lines of ICI had an ORR of 23% after rechallenge [111]. Zhao et al. evaluated 789 ICI rechallenges across multiple cancer types, with a pooled ORR and DCR after rechallenge of 43.1% and 71.9%, respectively, showing no significant difference compared with initial ICI treatment [108].

Selecting the patients that may benefit from a rechallenge with an ICI-based treatment while limiting the relapse of irAEs remains a hurdle. 

## 4. Discussion

Immune checkpoint inhibitors, in combination with tyrosine kinase inhibitors or other ICIs, have reached the frontline setting and significantly improved the prognosis for patients with metastatic clear cell renal cell carcinoma. This marks a major milestone in the treatment of mccRCC, but most patients will eventually discontinue first-line therapy due to several factors such as drug-related adverse events, treatment completion, complete response or maximum clinical benefit, pseudoprogression, systemic progression, and oligoprogression.

The most frequent reason for discontinuation in the pivotal trials for the current standard of care, namely nivolumab plus ipilimumab, pembrolizumab plus axitinib, pembrolizumab plus lenvatinib, and nivolumab plus cabozantinib, was disease progression, ranging from 51.9% of patients in the CheckMate 214 to 63.1% in the KEYNOTE 426 (Figure 2). Primary progressive patients appear to occur more frequently in patients treated with nivolumab + ipilimumab: 17.6% versus 5.4% to 11.3% of patients in IO-TKI combinations, but a direct comparison is unwarranted. Available data from trials in Table 1 suggest that antiangiogenic therapies are commonly used in later lines, with a preference for VEGFR inhibitors after IO-TKI combinations. Other options include PD-(L)1 inhibitors and mTOR inhibitors. PFS2 and OS data from clinical trials and retrospective studies suggest that second-line therapies provide significant benefits in terms of disease control and survival, but more research is needed to confirm these findings. Nivolumab plus ipilimumab after IO-TKI combinations have been evaluated in a small perspective trial with promising results, but previous experience with salvage ipilimumab has led to poor outcomes [112]. Retrospective data suggest that after nivolumab–ipilimumab as first-line treatments, TKI alone or in combination with ICIs is a viable option [57,103,104]. Data from the CONTACT-03 study showed a lack of benefit from adding ICI to cabozantinib monotherapy in the second or later line after progression to previous ICI treatment [97].

The second reason is the development of adverse events, ranging from 24.1% in the CheckMate 9ER to 36.9% in the CheckMate 214 (Figure 2). Patients who had to discontinue treatment for toxicity in the CheckMate 214 trial reportedly have a similar prognosis to other patients [106], whereas outcomes from trials evaluating outcomes after discontinuation for toxicity in IO-TKI combinations are not available. 

There are very few prospective data after the failure of immunotherapy combinations; therefore, clinicians face a complex decision-making process when it comes to managing second or subsequent lines of treatment in mccRCC patients. Different scenarios may require different approaches, such as continuing with ICI monotherapy or switching to targeted therapy or other ICI combinations. Pseudoprogression, in particular, can be challenging to differentiate from true progression, and the use of immune response (iRECIST) criteria can help guide treatment decisions, especially for ICI-ICI combinations. Increasing evidence supports local therapies for oligo-progression. 

In conclusion, the management of mRCC patients who have discontinued first-line therapy is a complex and evolving area that requires careful consideration of a range of elements. Further research is needed to better understand the optimal treatment approaches in this setting.

## Figures and Tables

**Table 2:** Reasons for discontinuation of both study drugs from the CONSORT diagrams.

	CheckMate 214: Nivolumab + Ipilimumab [26]	KEYNOTE-426: Pembrolizumab + Axitinib [27]	CheckMate 9ER: Nivolumab + Cabozantinib [31]	CLEAR: Lenvatinib + Pembrolizumab [33]
Median follow-up for OS at CONSORT diagram	67.7 months	30.6 months	32.9 months	26.6 months
Number of pts that discontinued therapy at last follow-up (% ^#^)	513 (547)	312 (429)	228 (320)	210 (352)
Disease progression (% ^#^)	266 (48.6)	197 (45.9) (181 radiologic + 16 clinical)	130 (40.6) (of which 1 per physician PET/CT confirmed PD, listed in others)	116 (33.0)
Treatment-related AEs (% ^#^)	148 (27.1)	78 (18.2)	32 (10)	Total discontinuations of both drugs for trAEs + unrelated AEs = 60 (17.0)
AEs unrelated to treatment (% ^#^)	40 (7.3)	/	24 (7.5)	Total discontinuations of both drugs for trAEs + unrelated AEs = 60 (17.0)
Maximum clinical benefit (% ^#^)	18 (3.3)	7 (1.6)	2 (0.6)	/
Withdrew consent or requested to discontinue treatment (% ^#^)	27 (4.9)	18 (4.2)	10 (3.1)	21 (4 withdrew consent, 17 patient choice) (6.0)
Died (% ^#^)	1 (1.8) ^^^	/	3 (0.9)	/
Lost to follow-up (% ^#^)	1 (1.8)	/	2 (0.6)	/
Other (% ^#^)	11 (2.0)	11 (2.6) various reasons: + 8 physician decision + 2 non-study anticancer therapy + 1 excluded medication	10 (3.1) various reasons: + 1 investigator decision + 2 underwent treatment for a new primary + 3 underwent local therapy/resection + 2 for poor status + 1 no longer measurable disease + 1 no target lesion	13 (3.7)
Poor compliance (% ^#^)	1 (1.8)	1 (0.2)	1 (0.3) (in others: patient refused to do anything)	/
Discontinued ICI per protocol completion (% ^#^)	Single drug discontinuation not allowed	19 (4.4)	Reported 14 (4.4) completion of treatment for both study drugs †	/

^#^ Expressed as a percentage of the safety population. † Per protocol, cabozantinib would be continued until progression or unacceptable toxicity; patients may have nivolumab per protocol and cabozantinib for other reasons. /: not reported in the CONSORT diagram. ^^^ Eight treatment-related deaths were first reported in the first paper in 2018 [17].

## Data Availability

The data is accessible and available from the cited papers.
